# Method for estimating high sdLDL-C by measuring triglyceride and apolipoprotein B levels

**DOI:** 10.1186/s12944-017-0417-6

**Published:** 2017-01-26

**Authors:** Toshiyuki Hayashi, Shinji Koba, Yasuki Ito, Tsutomu Hirano

**Affiliations:** 1 Department of Medicine, Division of Diabetes, Metabolism, and Endocrinology, Showa University School of Medicine, 1-5-8 Hatanodai, Shinagawa, Tokyo, 142-8666 Japan; 2 Department of Medicine, Division of Cardiology, Showa University School of Medicine, Shinagawa, Tokyo, Japan; 3 Reagent R&D Department, Denka Seiken Co., Ltd., Tokyo, Japan

**Keywords:** Apolipoprotein B, Coronary artery disease, Cholesterol, Small dense low-density lipoproteins, Triglycerides

## Abstract

**Background:**

We previously developed an assay to directly measure small dense (sd) low-density lipoprotein cholesterol (LDL-C) levels, which is not widely used in general clinical practice. Therefore, we propose a simpler method, “LDL window,” that uses conventional methods for estimating high sdLDL-C levels.

**Methods:**

We analyzed our previous studies (2006–2008) on healthy subjects and patients with type 2 diabetes and coronary artery disease (CAD). The sdLDL-C level was measured using the precipitation method, and LDL size was determined using gradient gel electrophoresis. The “LDL window” comprises the estimation of LDL particle number and size. We adopted apolipoprotein B (apoB) for the estimation of the LDL particle number and used 110 mg/dL as the cutoff value for hyper-apoB. Triglycerides (TGs) are a powerful inverse determinant of LDL particle size. Therefore, we adopted TG for the estimation of the LDL particle size and used 150 mg/dL as the cutoff value for hyper-TG. Subjects were stratified into the following four subgroups: normal, hyper-TG, hyper-apoB, and hyper-TG/-apoB. Non-high-density lipoprotein cholesterol (non-HDL-C) is a surrogate marker for apoB; therefore, the “alternative LDL window” comprised non-HDL-C (cutoff, 170 mg/dL) and TG.

**Results:**

The top quartile (Q4) of sdLDL-C (>31 mg/dL) doubled in patients with diabetes and CAD. The hyper-TG/-apoB group in the “LDL window” represented >90% Q4 and <4% Q1 and Q2, irrespective of the subjects. The sdLDL-C levels in the hyper-TG/-apoB group were 50% higher in patients with diabetes and CAD than those in controls. Similar results were obtained using the “alternative LDL window.”

**Conclusions:**

Our proposed “LDL window” may help identify patients at high risk of CAD independent of LDL-C.

**Electronic supplementary material:**

The online version of this article (doi:10.1186/s12944-017-0417-6) contains supplementary material, which is available to authorized users.

## Background

Low-density lipoprotein (LDL) is an atherogenic factor that can be fractionated into large buoyant (lb) and small dense (sd) particles according to size and density [1]. Abundant clinical evidence shows that sdLDL particles are more atherogenic than lbLDL particles [2–5], and the predominance of sdLDL incurs a three-fold increase in the risk for coronary artery diseases (CADs) [2–6]. The prospective Québec cardiovascular study revealed that a greater proportion of sdLDL, evaluated semi-quantitatively, is a significant and independent predictor of CAD [7]. Our group previously developed a simple precipitation assay for the selective and direct measurement of the sdLDL levels in serum or plasma [8] and demonstrated that not lbLDL-cholesterol (C) but the sdLDL-C levels were closely related to the angiographic and/or clinical severity of CAD independent of classical coronary risk factors [9, 10]. In a similar manner, using our method, a case-control analysis of the Framingham Offspring study revealed that the sdLDL-C/LDL-C ratio was significantly higher in subjects with CAD than that in those without CAD, regardless of sex [11]. Recent large cohort studies using our homogenous method [12] found that sdLDL-C is superior to conventional lipid measurements for predicting CAD risk [13–15]. However, the measurement of the sdLDL-C levels is not widely employed in general clinical practice.

Here we propose “LDL window” for estimating individuals with high sdLDL-C levels stratified according to the plasma triglyceride (TG) and apolipoprotein B (apo B) levels or non-high-density lipoprotein cholesterol (non-HDL-C).

## Methods

We developed a method using conventional lipid measurements to better facilitate the identification of individuals with high sdLDL-C levels and determined whether the significance of sdLDL-C is potentiated in patients with diabetes and CAD. The present study represents an analysis of our previous studies conducted during 2006–2008 that measured the sdLDL-C level and LDL size of healthy subjects (*n* = 1665) [16], patients with type 2 diabetes (*n* = 201), and patients with CAD without or with type 2 diabetes (*n* = 528, 354/174). Healthy subjects were employees of Denka Seiken Co. All patients with type 2 diabetes were diagnosed according to the criteria provided by the Japanese Diabetes Association. All patients with CAD exhibited significant coronary stenosis that was detected using coronary angiography. We excluded patients taking lipid-lowering agents [17–20]. During the previous study, patients with CAD or type 2 diabetes were not frequently treated using statins. Written informed consent was obtained from all individuals, and the local ethics committee approved the experimental protocol.

### Measurements

All plasma samples, which were obtained after the patients fasted overnight, were stored at –80 °C. The sdLDL-C level was measured using heparin–magnesium precipitation [8], and LDL particle size was measured using nondenaturing gradient-gel (2%–16%) electrophoresis (GGE) according to a published method [21]. Particles with diameters of <25.5 nm were determined of having the small-sized LDL phenotype, pattern B [22]. ApoB was measured using a turbidometric assay (Sekisui Kagaku Co.). Total-C, TG, LDL-C, HDL-C, and HbA1c levels were measured using standard laboratory procedures. lbLDL-C was estimated by subtracting the sdLDL-C level from the LDL-C level. Non-HDL-C was calculated by subtracting the HDL-C level from the total-C level. HbA1c (National Glycohemoglobin Standardization Program [NGSP]) values were estimated by 1.02 × HbA1c (Japan Diabetes Society[JDS]) + 0.25% [23].

### Quantile analysis of sdLDL-C

The sdLDL-C levels in healthy subjects (*n* = 1665) were stratified into quartiles (Q) as follows: Q1, <15.4 mg/dL; Q2, 15.4–21.9 mg/dL; Q3, 22.0–31.7 mg/dL; and Q4, >31.7 mg/dL. Patients with diabetes and CAD were similarly analyzed.

### Determination of hyper-apoB

**Fig. 1:**
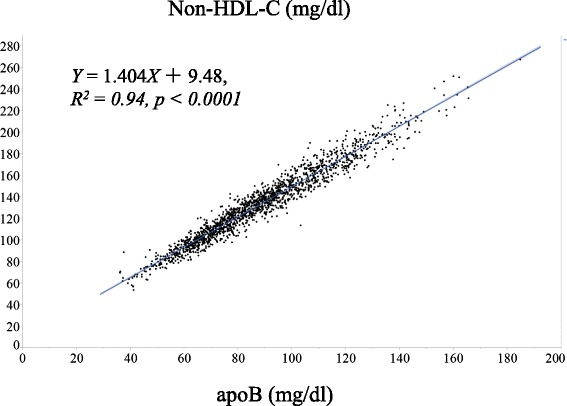
Correlations between non-HDL cholesterol (C) and apoB in healthy subjects (*n* = 1665). The curve-fitting parameters are *Y* = 1.404*X* + 9.48, *R* ^2^ = 0.94, *p* < 0.0001

The apoB levels correspond to non-HDL-C levels. The Japanese Atherosclerosis Society has not decided the designation of hyper-apoB. Therefore, we measured non-HDL-C, a surrogate marker for apoB [24], to determine the cutoff level of hyper-apoB. Non-HDL-C levels correlated highly with apoB levels in 1665 healthy subjects (*Y* = 1.404*X* + 9.48, *R* ^2^ = 0.94, *p* < 0.0001) (Fig. 1), and the value of high non-HDL-C (170 mg/dL) [24] corresponded to 113 mg/dL of apoB. Thus, we used 110 mg/dL as the cutoff value for hyper-apoB.

### Surrogate marker of LDL size

Small LDL particles were defined as those with an average diameter of <25.5 nm, the so-called pattern B [22]. TG is a powerful inverse determinant of LDL particle size [22]. In contrast, the sdLDL particle is cholesterol depleted. Therefore, the LDL-C/apoB ratio positively correlates with the diameter of the LDL particle [25]. When we compared whether the TG or LDL-C/apoB ratio more strongly correlated with LDL particle diameters, we found that TG is more strongly correlated with LDL size (*r* = –0.52 vs. 0.44 data not shown). Therefore, we selected TG as a surrogate marker for LDL size. The TG/HDL-C ratio strongly correlates with LDL diameter [26]. However, we could not detect an additive effect of TG/HDL-C on improving the correlation with LDL size beyond TG (data not shown).

### The “LDL window” and the “alternative LDL window”

**Fig. 2:**
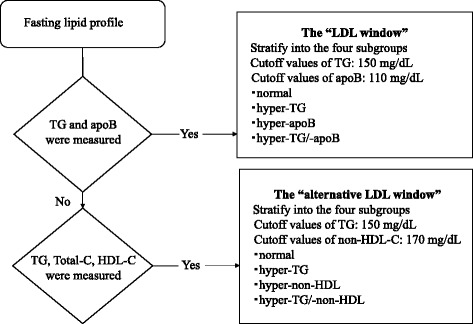
The flowchart of the use of the “LDL window” and the “alternative LDL window” for estimating high sdLDL-C

We proposed the “LDL window” and the “alternative LDL window” for estimating high sdLDL-C. Figure 2 shows its usage. The “LDL window” comprises the number and sizes of LDL particles estimated according to the plasma apoB and TG levels, respectively. The cutoff values of the hyper-apoB and -TG levels were 110 and 150 mg/dL, respectively. Subjects were stratified into the subgroups as follows: normal (apoB <110 and TG <150 mg/dL), hyper-TG (apoB <110 and TG ≥150 mg/dL), hyper-apoB (apoB ≥110 and TG <150 mg/dL), and hyper-TG/-apoB (apoB ≥110 and TG ≥150 mg/dL).

The “alternative LDL window” comprises the number and sizes of LDL particles estimated according to the plasma non-HDL-C and TG levels.

Non-HDL-C is a surrogate marker for apoB [22]; therefore, it was used instead of apoB, and subjects were grouped as follows: normal (non-HDL-C <170 and TG <150 mg/dL), hyper-TG (non-HDL-C <170 and TG ≥150 mg/dL), hyper-non-HDL (non-HDL-C ≥170 and TG <150 mg/dL), and hyper-TG/-non-HDL (non-HDL-C ≥170 and TG ≥150 mg/dL).

### Statistical analysis

Data are expressed as mean ± standard deviation (SD). Statistical analyses were performed using JMP 11.0 (SAS Institute, Cary, NC, USA). Comparisons among multiple groups were performed using one-way analysis of variance, and statistical significance was evaluated using the Bonferroni–Dunn post-hoc test. Pearson’s linear regression analysis was used to evaluate the relationship between the apoB and non-HDL-C levels in healthy subjects. Significance was defined as *p* < 0.05.

## Results

Table 1 shows the general and metabolic characteristics of all subjects. The mean age of patients with CAD was greater than that of healthy subjects and patients with diabetes. Among patients with CAD, 33% were diagnosed with diabetes. TG levels were significantly higher in patients with CAD and diabetes than those in healthy subjects. HDL-C levels were lower in patients with CAD or diabetes than those in healthy subjects. LDL-C, non-HDL-C, and apoB levels were higher in patients with CAD than those in healthy subjects and were further elevated in patients with diabetes. The mean plasma sdLDL-C levels and sdLDL-C/LDL-C ratios were 25 mg/dL and 21.6%, respectively, in the healthy subjects. The sdLDL-C levels were higher in patients with CAD than those in healthy subjects and were further increased in patients with diabetes. The lbLDL-C levels were lower in patients with CAD than those in healthy subjects. The mean LDL size was smaller in patients with diabetes than that in healthy subjects and even smaller than that in patients with CAD. The LDL-C/apoB ratios were significantly lower in patients with CAD or diabetes than those in healthy subjects, which was consistent with the difference in LDL size.

**Table 1:** General and metabolic characteristics of the healthy subjects, patients with coronary artery disease (CAD), and patients with type 2 diabetes (Diabetes)

	Healthy subjects	CAD	Diabetes
Number	1665	528	201
Age, years	41 ± 12	67 ± 11^a^	61 ± 12^a,b^
Sex, M/F	954/711	414/114	125/76
Diabetes, n (%)	0	174 (33)	201 (100)
Total cholesterol, mg/dl	195.5 ± 33.5	197.1 ± 36.4	205.3 ± 49.6^a,b^
HDL-C, mg/dl	62.0 ± 15.3	47.2 ± 13.5^a^	48.2 ± 13.3^a^
TG, mg/dl	95.0 ± 65.2	131.5 ± 76.1^a^	142.0 ± 85.1^a^
LDL-C, mg/dl	113.9 ± 31.6	119.6 ± 32.4^a^	129.2 ± 40.4^a,b^
sdLDL-C, mg/dl	25.0 ± 13.0	35.3 ± 20.5^a^	40.3 ± 24.9^a,b^
lbLDL-C, mg/dl	88.9 ± 24.9	84.3 ± 29.1^a^	88.9 ± 31.7
Non-HDL-C, mg/dl	133.4 ± 33.7	149.9 ± 35.5^a^	157.1 ± 47.4^a,b^
sdLDL-C/LDL-C, %	21.6 ± 8.3	29.6 ± 15.0^a^	30.5 ± 14.5^a^
ApoB, mg/dl	88.3 ± 23.4	98.0 ± 23.6^a^	105.3 ± 31.4^a,b^
LDL-C/apoB	1.29 ± 0.12	1.22 ± 0.17^a^	1.23 ± 0.12^a^
LDL size, nm	26.1 ± 0.51	25.6 ± 0.41^a^	25.8 ± 0.50^a,b^

Data are expressed as mean ± standard deviation

Significance at *p* < 0.0001–0.05 by ANOVA

*CAD* coronary artery disease, *HDL-C* high-density lipoprotein-cholesterol, *TG* triglyceride, *LDL-C* low-density lipoprotein-cholesterol, *sdLDL-C* small dense LDL-C, *lbLDL-C* large buoyant LDL-C, *Non-HDL-C* non high-density lipoprotein-cholesterol, *Apo* apolipoprotein

^a^vs. Healthy subjects; ^b^vs. CAD

Table 2 lists lipid profiles of healthy subjects stratified according to their sdLDL-C quartile. The total-C, TG, LDL-C, lbLDL-C, non-HDL-C, and apoB levels and sdLDL-C/LDL-C ratios were significantly increased, and LDL sizes decreased with the increase in the sdLDL-C quartile. The LDL/apoB ratios were similar between Q1 and Q3 and significantly decreased in Q4. The representations of the small-LDL phenotype (pattern B) in Q4 were 36.9 and 2.4% in Q1.

**Table 2:** Lipid measurements stratified by sdLDL-C quartiles in 1665 healthy subjects

sdLDL-C quartile	Q1	Q2	Q3	Q4
	(<15.4 mg/dl)	(15.4–21.9 mg/dl)	(22.0–31.7 mg/dl)	(>31.7 mg/dl)
Number	415	416	417	417
(M/F)	(182/233)	(222/194)	(237/180)	(313/104)
Total cholesterol, mg/dl	171.8 ± 25.8	187.2 ± 28.1^a^	200.0 ± 27.9^a,b^	222.8 ± 29.4^a,b,c^
HDL-C, mg/dl	67.1 ± 15.0	65.3 ± 15.2	61.5 ± 14.6^a,b^	54.4 ± 13.4^a,b,c^
TG, mg/dl	67.9 ± 39.4	75.4 ± 37.4^a^	90.0 ± 49.4^a,b^	146.6 ± 88.4^a,b,c^
LDL-C, mg/dl	88.1 ± 21.1	104.2 ± 22.6^a^	120.6 ± 24.8^a,b^	142.6 ± 28.0^a,b,c^
sdLDL-C, mg/dl	11.7 ± 2.5	18.7 ± 1.94^a^	26.2 ± 2.7^a,b^	43.3 ± 10.4^a,b,c^
lbLDL-C, mg/dl	76.4 ± 20.4	85.6 ± 22.3^a^	94.4 ± 24.2^a,b^	99.4 ± 26.0^a,b,c^
Non-HDL-C, mg/dl	104.7 ± 20.9	121.9 ± 22.4^a^	138.5 ± 25.4^a,b^	168.4 ± 27.4^a,b,c^
sdLDL-C/LDL-C, %	13.9 ± 4.0	18.7 ± 4.5^a^	22.6 ± 5.1^a,b^	31.0 ± 7.4^a,b,c^
ApoB, mg/dl	67.3 ± 13.5	79.8 ± 14.3^a^	92.0 ± 16.6^a,b^	113.8 ± 18.5^a,b,c^
LDL-C/apoB	1.30 ± 0.12	1.30 ± 0.11	1.31 ± 0.10	1.25 ± 0.14^a,b,c^
LDL size, nm	26.3 ± 0.33	26.2 ± 0.48^a^	26.1 ± 0.43^a,b^	25.7 ± 0.58^a,b,c^
Pattern B, n (%)	10 (2.4)	20 (4.8)	59 (14.2)	154 (36.9)

Data are expressed as mean ± standard deviation

*HDL-C* high-density lipoprotein-cholesterol, *TG* triglycerides, *LDL-C* low-density lipoprotein-cholesterol, *sdLDL-C* small dense LDL-C, *lbLDL-C* large buoyant LDL-C, *Non-HDL-C* non high-density lipoprotein-cholesterol, *Apo* apolipoprotein, *Pattern B* LDL phenotype with an average diameter of <25.5 nm

Significance at *p* < 0.0001–0.05 by ANOVA

^a^vs. Q1; ^b^vs. Q2; ^c^vs. Q3

Table 3 lists the characteristics and lipid measurements of healthy subjects in the “LDL window.” The majority (75.7%) of healthy subjects were stratified into the normal group and 6.5% were included in the hyper-TG/-apoB group. The hyper-TG/-apoB group had the lowest HDL-C levels and smallest LDL size. The representation of pattern B in the hyper-TG/-apoB group was 56.5%, and the normal group represented 6.4%. Total-C levels were similar between the hyper-apoB and hyper-TG/-apoB groups. The LDL-C and lbLDL-C levels were the highest in the hyper-apoB group, whereas the sdLDL-C levels were the highest in the hyper-TG/-apoB group. The sdLDL-C levels were 1.5-fold higher in the hyper-TG group, 2-fold higher in the hyper-apoB group, and 2.5-fold higher in the TG/hyper-apoB group than those in the normal group. The hyper-TG/-apoB represented 90.7% of Q4 and 0.9% and 2.8% of Q1 and Q2, respectively.

**Table 3:** LDL window in 1665 healthy subjects

	“LDL window”			
Groups	Normal	Hyper-TG	Hyper-apoB	Hyper-TG/-apoB
Number (M/F)	1261 (617/644)	100 (86/14)	196 (152/43)	108 (98/10)
%N/total N	75.7	6.0	11.8	6.5
Total cholesterol, mg/dl	185.4 ± 27.5	190.9 ± 23.0	239.9 ± 22.5^a,b^	236.3 ± 24.5^a,b^
HDL-C, mg/dl	64.7 ± 15.1	50.3 ± 12.0^a^	57.7 ± 13.1^a,b^	48.8 ± 10.0^a,c^
TG, mg/dl	72.3 ± 28.6	224.1 ± 89.7^a^	102.8 ± 26.7^a,b^	225.9 ± 93.2^a,c^
LDL-C, mg/dl	103.6 ± 23.9	108.1 ± 22.2	162.2 ± 19.0^a,b^	151.3 ± 25.2^a,b,c^
sdLDL-C, mg/dl	20.4 ± 8.9	31.2 ± 10.8^a^	37.2 ± 11.1^a,b^	49.2 ± 14.6^a,b,c^
lbLDL-C, mg/dl	83.1 ± 20.4	76.9 ± 20.4^a^	125.0 ± 19.5^a,b^	102.1 ± 23.5^a,b,c^
Non-HDL-C, mg/dl	120.6 ± 24.9	140.6 ± 19.8^a^	182.2 ± 16.8^a,b^	187.5 ± 23.3^a,b^
sdLDL-C/LDL-C, %	19.7 ± 7.0	29.3 ± 9.6^a^	23.0 ± 6.7^a,b^	32.8 ± 9.0^a,b,c^
ApoB, mg/dl	78.9 ± 16.0	93.9 ± 13.1^a^	122.6 ± 10.5^a,b^	128.8 ± 14.2^a,b,c^
LDL-C/apoB	1.30 ± 0.10	1.14 ± 0.16^a^	1.32 ± 0.09^b^	1.17 ± 0.12^a,c^
LDL size, nm	26.2 ± 0.41	25.5 ± 0.73^a^	25.8 ± 0.41^a,b^	25.4 ± 0.52^a,c^
Pattern B, n (%)*	81 (6.4)	46 (46)	55 (28.1)	61 (56.5)
sdLDL-C Q1, Q2, Q3, Q4, n	407, 391, 328, 135	6, 14, 27, 53	1, 8, 56, 131	1, 3, 6, 98
sdLDL-C Q1, Q2, Q3, Q4, %	32.3, 31.0, 26.0, 10.7	6.0, 14.0, 27.0, 53.0	0.5, 4.1, 28.6, 66.8	0.9, 2.8, 5.6, 90.7

Data are expressed as mean ± standard deviation

*HDL-C* high-density lipoprotein-cholesterol, *TG* triglycerides, *LDL-C* low-density lipoprotein-cholesterol, *sdLDL-C* small dense LDL-C, *lbLDL-C* large buoyant LDL-C, *Non-HDL-C* non high-density lipoprotein-cholesterol, *Apo* apolipoprotein, *Pattern B* LDL phenotype with an average diameter of <25.5 nm

Significance at *p* < 0.0001–0.05 by ANOVA

*Statistically significant differences

^a^vs. normal; ^b^hyper-TG; ^c^vs. hyper-apoB

Table 4 lists the characteristics and lipid measurements of patients with type 2 diabetes in the “LDL window.” Approximately 20% of the patients with type 2 diabetes were classified into the hyper-TG/-apoB group, which was 3-fold higher than the percentage of healthy subjects. The total-C, TG, non-HDL-C, and apoB levels were the highest and LDL sizes were the smallest in the hyper-TG/-apoB group. The prevalence of pattern B was 61.5 and 17.1% in the hyper-TG/-apoB and normal groups, respectively. The LDL-C levels were similar between the hyper-apoB and the hyper-TG/-apoB groups. The lbLDL-C levels were the highest in the hyper-apoB group, whereas the sdLDL-C levels were the highest in the hyper-TG/-apoB group. The sdLDL-C levels in the hyper-TG/-apoB group were 2.8-fold higher than those in the normal group. The Q4 of sdLDL-C distributed in the hyper-TG/-apoB was 92.3 and 0% in Q1 and Q2.

**Table 4:** LDL window in patients with type 2 diabetes

	“LDL window”			
Groups	Normal	Hyper-TG	Hyper-apoB	Hyper-TG/-apoB
Number (M/F)	105 (66/39)	19 (12/7)	38 (22/16)	39 (25/14)
%N/total N	52.2	9.5	18.9	19.4
Total cholesterol, mg/dl	176.0 ± 28.6	178.4 ± 24.8	241.7 ± 38.9^a,b^	261.4 ± 40.7^a,b,c^
HDL-C, mg/dl	49.3 ± 13.2	39.8 ± 7.9^a^	51.8 ± 14.5^b^	45.7 ± 12.6
TG, mg/dl	96.0 ± 26.5	198.4 ± 48.3^a^	118.0 ± 21.7^b^	261.3 ± 109.3^a,b,c^
LDL-C, mg/dl	105.6 ± 23.6	104.4 ± 20.6	168.9 ± 31.0^a,b^	165.8 ± 33.4^a,b^
sdLDL-C, mg/dl	26.4 ± 11.8	38.2 ± 17.2^a^	45.7 ± 18.9^a^	73.0 ± 27.1^a,b,c^
lbLDL-C, mg/dl	79.2 ± 20.1	66.1 ± 24.5	123.2 ± 34.7^a,b^	92.7 ± 31.8^a,b,c^
Non-HDL-C, mg/dl	126.7 ± 25.8	138.7 ± 22.5	189.9 ± 32.2^a,b^	215.7 ± 37.5^a,b,c^
sdLDL-C/LDL-C, %	24.9 ± 9.5	37.1 ± 15.8^a^	27.7 ± 13.0^b^	44.6 ± 15.8^a,c^
ApoB, mg/dl	84.6 ± 16.5	93.9 ± 13.8	128.4 ± 20.0^a,b^	143.7 ± 25.5^a,b,c^
LDL-C/apoB	1.24 ± 0.09	1.10 ± 0.12^a^	1.31 ± 0.09^a,b^	1.15 ± 0.13^a,c^
LDL size, nm	25.9 ± 0.44	25.6 ± 0.42	25.9 ± 0.49	25.4 ± 0.52^a,c^
Pattern B, n (%)*	18 (17.1)	6 (31.6)	11 (29.0)	24 (61.5)
sdLDL-C Q1, Q2, Q3, Q4, n	19, 21, 33, 32	1, 1, 6, 11	1, 3, 7, 27	0, 0, 3, 36
sdLDL-C Q1, Q2, Q3, Q4, %	18.1, 20.0, 31.4, 30.5	5.3, 5.3, 31.6, 57.9	2.6, 7.9, 18.4, 71.1	0, 0, 7.7, 92.3

Data are expressed as mean ± standard deviation

*HDL-C* high-density lipoprotein-cholesterol, *TG* triglycerides, *LDL-C* low-density lipoprotein-cholesterol, *sdLDL-C* small dense LDL-C, *lbLDL-C* large buoyant LDL-C, *Non-HDL-C* non high-density lipoprotein-cholesterol, *Apo* apolipoprotein, *Pattern B* LDL phenotype with an average diameter of <25.5 nm

Significance at *p* < 0.0001–0.05 by ANOVA

*Statistically significant differences

^a^vs. normal; ^b^hyper-TG; ^c^vs. hyper-apoB

Table 5 shows the characteristics and lipid measurements of patients with CAD in the “LDL window.” Approximately 40% of these patients were classified into the hyper-TG/-apoB group. The total-C and LDL-C levels were similar between the hyper-apoB and hyper-TG/-apoB groups. Non-HDL-C and apoB levels were the highest in the hyper-TG/-apoB group. LDL sizes were smaller in the hyper-TG and hyper-TG/-apoB groups than those in the normal group. The frequencies of pattern B were 77.6 and 80.5% in the hyper-TG and hyper-TG/-apoB groups, respectively, and were 35.6 and 39.7% in the normal and hyper-apoB groups, respectively. The lbLDL-C levels were the highest in the hyper-apoB group, whereas the sdLDL-C levels were the highest in the hyper-TG/-apoB group. The sdLDL-C levels in the hyper-TG/-apoB group were 2.3-fold higher than those in the normal group. The Q4 of sdLDL-C included 96.1% of the hyper-TG/-apoB group and 0% in Q1 and Q2. The sdLDL-C levels and prevalance of the hyper-apoB/-TG group were not different between patients with CAD with and without diabetes (data not shown).

**Table 5:** LDL window in 528 patients with CAD including diabetes

	“LDL window”			
Groups	Normal	Hyper-TG	Hyper-apoB	Hyper-TG/-apoB
Number (M/F)	312 (244/68)	76 (67/9)	63 (46/17)	77 (57/20)
%N/total N	59.1	14.4	11.9	14.6
Diabetes, n (%)	102 (32.6)	23 (30.2)	20 (31.7)	29 (37.6)
Total cholesterol, mg/dl	180.7 ± 27.2	191.6 ± 22.8^a^	232.9 ± 29.7^a,b^	239.4 ± 29.7^a,b^
HDL-C, mg/dl	49.8 ± 14.3	43.7 ± 12.3^a^	46.1 ± 11.7	40.7 ± 8.8^a^
TG, mg/dl	92.1 ± 28.1	222.6 ± 86.1^a^	104.6 ± 32.1^b^	223.1 ± 70.3^a,c^
LDL-C, mg/dl	106.5 ± 22.9	105.9 ± 20.0	159.5 ± 25.7^a,b^	153.3 ± 28.3^a,b^
sdLDL-C, mg/dl	26.6 ± 13.9	37.6 ± 14.6^a^	44.1 ± 24.6^a^	60.8 ± 19.0^a,b,c^
lbLDL-C, mg/dl	79.9 ± 23.0	68.3 ± 23.9^a^	115.3 ± 33.5^a,b^	92.4 ± 31.5^a,b,c^
Non-HDL-C, mg/dl	130.9 ± 22.2	147.9 ± 20.8^a^	186.8 ± 25.9^a,b^	198.6 ± 26.5^a,b,c^
sdLDL-C/LDL-C, %	25.4 ± 13.3	36.5 ± 15.0^a^	28.0 ± 15.2^b^	40.8 ± 13.4^a,c^
ApoB, mg/dl	85.0 ± 14.3	94.5 ± 11.6^a^	124.4 ± 13.6^a,b^	132.2 ± 16.5^a,b,c^
LDL-C/apoB	1.25 ± 0.16	1.11 ± 0.15^a^	1.28 ± 0.14^b^	1.15 ± 0.13^a,c^
LDL size, nm	25.7 ± 0.36	25.3 ± 0.43^a^	25.7 ± 0.32^b^	25.4 ± 0.40^a,c^
Pattern B, n (%)*	111 (35.6)	59 (77.6)	25 (39.7)	62 (80.5)
sdLDL-C Q1, Q2, Q3, Q4, n	61, 75, 81, 95	3, 5, 23, 45	3, 5, 13, 42	0, 0, 3, 74
sdLDL-C Q1, Q2, Q3, Q4, %	19.6, 24.0, 26.0, 30.5	4.0, 6.6, 30.3, 59.2	4.8, 7.9, 20.6, 66.7	0, 0, 3.9, 96.1

Data are expressed as mean ± standard deviation

*HDL-C* high-density lipoprotein-cholesterol, *TG* triglycerides, *LDL-C* low-density lipoprotein-cholesterol, *sdLDL-C* small dense LDL-C, *lbLDL-C* large buoyant LDL-C, *Non-HDL-C* non high-density lipoprotein-cholesterol, *Apo* apolipoprotein, *Pattern B* LDL phenotype with an average diameter of <25.5 nm

Significance at *p* < 0.0001–0.05 by ANOVA

*Statistically significant differences

^a^vs. normal; ^b^hyper-TG; ^c^vs.hyper-apoB

**Fig. 3:**
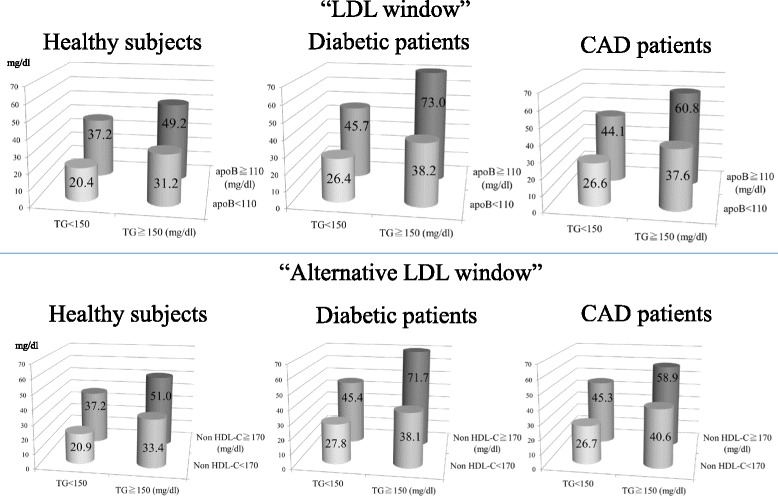
The sdLDL-C levels stratified by the “LDL window” and the “alternative LDL window” of healthy subjects, patients with diabetes, and patients with CAD. Top panel: small dense (sd) LDL-C levels stratified by triglycerides (TG) and apolipoprotein B (apoB) in healthy subjects (*n* = 1665), patients with diabetes (*n* = 201), and patients with CAD (*n* = 528). The “LDL window” comprises the groups as follows: normal (apoB <110 mg/dL and TG <150 mg/dL), hyper-TG (apoB <110 mg/dL and TG ≥150 mg/dL), hyper-apoB (apoB ≥110 mg/dL and TG <150 mg/dL), and hyper-TG/-apoB (apoB ≥110 mg/dL and TG ≥150 mg/dL). Bottom panel: sdLDL-C levels stratified by TG and non-HDL-C in healthy subjects (*n* = 1665), patients with diabetes (*n* = 201), and patients with CAD (*n* = 528). The “alternative LDL window” comprises the groups as follows: normal (non-HDL-C <170 mg/dL and TG <150 mg/dL), hyper-TG (non-HDL-C <170 mg/dL and TG ≥150 mg/dL), hyper-non-HDL (non-HDL-C ≥170 mg/dL and TG <150 mg/dL), and hyper-TG/-non-HDL (non-HDL-C ≥170 mg/dL and TG ≥150 mg/dL).

The top panel of Fig. 3 depicts the sdLDL-C levels stratified by the “LDL window” of healthy subjects, patients with diabetes, and patients with CAD according to the data presented in Tables 3, 4, and 5. The sdLDL-C levels were significantly higher in the hyper-TG/-apoB group independent of subjects’ characteristics, and these levels were significantly higher in patients with diabetes or CAD than those in healthy subjects. The bottom panel of Fig. 3 shows the sdLDL-C levels stratified by the “alternative LDL window” according to non-HDL-C and TG levels in healthy subjects and patients with diabetes or CAD. The general and metabolic characteristics of subjects list are shown in the Additional file 1: Table S1, S2 and S3. The characteristics of subjects stratified by non-HDL-C and TG levels were comparable to those stratified by apoB and TG levels. The sdLDL-C levels were significantly higher in the hyper-TG/-apoB group than those in the other groups, irrespective of subjects’ characteristics. Furthermore, these levels were significantly higher in patients with diabetes and CAD than those in healthy subjects. We compared the "LDL window" and the "alternative LDL window" for healthy controls and diabetic and CAD groups. There were no significant differences between the "LDL window" and the "alternative LDL window" in the distribution of patients and sdLDL-C levels.

## Discussion

The sdLDL particles may be highly atherogenic because of their greater ability to penetrate the arterial wall, their lower affinity for the LDL receptor, their prolonged half-life in plasma, and their lower resistance to oxidative stress than the lbLDL particles [1, 3]. We developed the precipitation [8] and homogenous methods [12] for measuring sdLDL-C. Our direct homogenous sdLDL-C assays were employed by large cohort studies [13–15], which revealed that the sdLDL-C levels more sensitively predict CAD events than lbLDL-C or LDL-C levels. Because the homogenous method was developed using the precipitation method as a reference, the sdLDL-C levels determined by the two methods should be identical [12], and the results acquired using each method excellently correlated with ultracentrifugation methods [12]. Thus, there should be no difference in interpreting the data acquired using each assay, and the data presented here shows that using the precipitation method is applicable to the homogenous method.

Measurement of LDL size and determination of the prevalence of the sdLDL phenotype (Pattern B) is important for evaluating the residual risk independent of the LDL-C level. LDL size is typically small in individuals with severe hypertriglyceridemia such as chylomicronemia, although they are not considered at a high risk for CAD [27, 28]. In contrast, individuals with moderate hypertriglyceridemia accompanied by hypercholesterolemia, including those with familial combined hyperlipidemia [16] as well as many patients with type 2 diabetes, are at a high risk for CAD. These observations suggest that quantification of sdLDL (sdLDL-C level) is a more sensitive marker for risk than qualification of LDL (LDL size) when predicting CAD.

The “LDL window” comprises LDL particle number and size. A single molecule of apoB is the principal structural component of the lipoprotein particles of very-low-density lipoprotein (VLDL), intermediated-density lipoprotein (IDL), and LDL. The long plasma residence time of LDL compared with that of VLDL or IDL accounts for the association of ≥90% of plasma apoB with LDL [29]. Therefore, the plasma apoB level approximates the LDL particle number as revealed by nuclear magnetic resonance spectroscopy (*r* ^*2*^ = 0.79) [30]. However, this estimation is not applicable to patients with hypertriglyceridemia because TG-rich lipoprotein apoB is contaminated with plasma apoB. The discordance (LDL particle number/ApoB) is not associated with plasma TG levels. Moreover, the discordance (LDL-particle number > apoB) increases with greater insulin resistance, suggesting that apoB underestimates LDL particle number in patients with insulin resistance that is typically accompanied by hypertriglyceridemia. Despite these critical limitations, we conclude that apoB serves as the best marker for LDL particle number among conventional analytical methods.

The prevalence of the hyper-apoB/-TG group was only 6.5% in the healthy subjects studied here, and this group in the “LDL window” represented 91% of sdLDL-C Q4 and 1% of Q1, indicating efficient detection of subjects with high sdLDL-C levels. The sdLDL-C level was higher in the hyper-apoB group than that in the hyper-TG group, suggesting that an increase in LDL particle number, rather than the decrease of LDL size, more strongly contributed to the elevated sdLDL-C level.

Patients with type 2 diabetes often have hypertriglyceridemia and mild elevation of LDL-C, both of which are associated with a high prevalence of CAD events [31]. We found that the elevation of LDL-C in diabetic populations was solely attributable to increased sdLDL-C [32]. Here we found that 20% of diabetic patients were classified into the hyper-apoB/-TG group, which was 3-fold higher than healthy subjects. The hyper-apoB/-TG group represented 92% of sdLDL-C Q4 and 0% of Q1 and Q2, indicating an increased discrimination between higher sdLDL-C levels in patients with diabetes compared with healthy subjects. SdLDL-C values in the hyper-apoB/-TG group were increased by 50% compared with those in the same group of healthy subjects (73 mg/dL vs. 49 mg/dL, *p* < 0.0001). Thus, the “LDL window” efficiently estimated very high sdLDL-C levels, particularly in subjects with diabetes. The high prevalence of sdLDL particles has been documented in patients with CAD [33–36]. Similar to patients with diabetes, a high prevalence of the hyper-apoB/-TG group was observed in patients with CAD who also had sdLDL-C levels that were 50% higher than those of the same group of healthy subjects. There was no significant difference in the sdLDL-C levels and prevalence of the hyper-apoB/-TG group between patients with CAD with and without diabetes, suggesting that diabetes is not an obligate factor and that other mechanisms must be involved in causing the hyper-apoB/-TG phenotype in patients with CAD.

We proposed the “LDL window” stratified by apoB and TG, but apoB is not commonly measured in general practice. Therefore, we defined the “alternative LDL window” that incorporates non-HDL-C instead of apoB. Finally, we obtained comparable results between two “LDL windows.” The “alternative LDL window” was particularly useful for identifying individuals with high sdLDL-C levels in earlier studies lacking apoB data. For example, the Suita study reveals that the prevalence of CAD is markedly higher among individuals with hyper-TG (>150 mg/dL) and -non-HDL-C (>190 mg/dL) [37]. According to our “alternative LDL window,” it is highly likely that these subjects had high sdLDL-C levels. Therefore, the “LDL window” may be useful for identifying groups with high sdLDL-C levels in previous literatures.

## Conclusions

The “LDL window” identifies individuals with high sdLDL-C levels. Therefore, we recommend that more attention should be paid when TG and apoB (or non-HDL-C) levels are high, particularly for patients with diabetes and those with CAD, because their sdLDL-C levels are likely to be substantially elevated.

## Additional file

Additional file 1: Table S1. Alternative LDL window in healthy subjects. **Table S2.** Alternative LDL window in patients with type 2 diabetes. **Table S3.** Alternative LDL window in 528 patients with CAD including diabetes. (DOCX 29 kb)

## Abbreviations

apo: Apolipoprotein
CAD: Coronary artery disease
HDL-C: High-density lipoprotein-cholesterol
lbLDL-C: Large buoyant LDL-C
LDL-C: Low-density lipoprotein-C
non-HDL-C: Non-high-density lipoprotein cholesterol
sd LDL-C: Small dense LDL-C
SD: Standard deviation
TG: Triglyceride
