# Supplementary material for: Method for estimating high sdLDL-C by measuring triglyceride and apolipoprotein B levels
Source: Lipids Health Dis. 2017 Jan 26;16:21. doi: 10.1186/s12944-017-0417-6 (PMC5270205; doi:10.1186/s12944-017-0417-6)
Supplement: Additional file 1: Table S1. — Alternative LDL window in healthy subjects. Table S2. Alternative LDL window in patients with type 2 diabetes. Table S3. Alternative LDL window in 528 patients with CAD including diabetes. (DOCX 29 kb) [file 12944_2017_417_MOESM1_ESM.docx]

**Additional file 1**

**Table S1. Alternative LDL window in healthy subjects**

|  | “Alternative LDL window” | | | |
| --- | --- | --- | --- | --- |
| Groups | Normal | Hyper-TG | Hyper-nonHDL | Hyper-TG/-non-HDL |
| Number (M/F)  %N/total N | 1294 (652/642) | 123 (107/16) | 163 (118/45) | 85 (77/8) |
|  | 77.7 | 7.4 | 9.8 | 5.1 |
| Total cholesterol, mg/dl | 186.1 ± 27.4 | 194.6 ± 22.4^a^ | 245.9 ± 21.0^a,b^ | 234.4 ± 22.6^a,b^ |
| HDL-C, mg/dl | 64.4 ± 15.1 | 50.3 ± 12.0^a^ | 59.1 ± 14.0^a,b^ | 48.5 ± 9.5^a,c^ |
| TG, mg/dl | 73.4 ± 29.0 | 216.5 ± 71.3^a^ | 200.9 ± 28.0^a,b^ | 237.5 ± 113.7^a,b,c^ |
| LDL-C, mg/dl | 104.6 ± 24.4 | 112.3 ± 22.0^a^ | 166.6 ± 17.7^a,b^ | 157.0 ± 25.4^a,b,c^ |
| sdLDL-C, mg/dl | 20.9 ± 9.4 | 33.4 ± 11.7^a^ | 37.2 ± 11.2^a,b^ | 51.0 ± 15.0^a,b,c^ |
| lbLDL-C, mg/dl | 83.7 ± 20.5 | 78.9 ± 19.7 | 129.4 ± 17.6^a,b^ | 106.1 ± 24.2^a,b,c^ |
| Non-HDL-C, mg/dl | 121.6 ± 24.7 | 244.2 ± 19.3^a^ | 186.8 ± 14.8^a,b^ | 194.9 ± 20.9^a,b,c^ |
| sdLDL-C/LDL-C, % | 20.0 ± 7.2 | 30.0 ± 9.6^a^ | 22.3 ± 6.3^a,b^ | 32.8 ± 9.1^a,b,c^ |
| ApoB, mg/dl | 80.0 ± 17.0 | 98.4 ± 15.1^a^ | 123.6 ± 11.5^a,b^ | 131.8 ± 15.0^a,b,c^ |
| LDL-C/apoB | 1.30 ± 0.10 | 1.14 ± 0.15^a^ | 1.35 ± 0.09^a,b^ | 1.19 ± 0.13^a,b,c^ |
| LDL size, nm | 262.0 ± 4.2 | 255.5 ± 7.0^a^ | 259.2 ± 4.2^a,b^ | 255.0 ± 5.4^a,c^ |
| Pattern B, n (%) | 96 (7.4) | 61 (49.6) | 40 (24.5) | 46 (54.1) |
| sdLDL-C Q1, Q2, Q3, Q4, n | 408, 392, 335, 159 | 7, 14, 28, 74 | 0, 7, 49, 107 | 0, 3, 5, 77 |
| sdLDL-C Q1, Q2, Q3, Q4, % | 31.5, 31.3, 25.9, 12.3 | 5.7, 11.4, 22.8, 60.2 | 0, 4.3, 30.1, 65.6 | 0, 3.5, 5.9, 90.6 |

Data are expressed as mean ± standard deviation

Significance at p < 0.0001–0.05 by ANOVA

^a^, vs. normal; ^b^, hyper-TG; ^c^, vs. hyper-nonHDL

HDL-C: high-density lipoprotein-cholesterol, TG: triglycerides, LDL-C: low-density lipoprotein-cholesterol, sdLDL-C: small dense LDL-C, lbLDL-C: large buoyant LDL-C, Non-HDL-C: non high-density lipoprotein-cholesterol, Apo: apolipoprotein, Pattern B: LDL phenotype with an average diameter

**Table S2. Alternative LDL window in patients with type 2 diabetes**

|  | “Alternative LDL window” | | | |
| --- | --- | --- | --- | --- |
| Groups | Normal | Hyper-TG | hyper-nonHDL | hyper-TG/-non-HDL |
| Number (M/F)  %N/total N | 113 (71/42) | 21 (13/8) | 25 (13/12) | 42 (28/14) |
|  | 56.2 | 10.4 | 12.4 | 20.9 |
| Total cholesterol, mg/dl | 178.0 ± 28.8 | 182.4 ± 27.0 | 257.2 ± 37.4^a,b^ | 259.4 ± 40.0^a,b^ |
| HDL-C, mg/dl | 48.8 ± 13.1 | 41.2 ± 9.6 | 56.6 ± 15.0^a,b^ | 45.0 ± 11.7^c^ |
| TG, mg/dl | 98.0 ± 26.8 | 196.5 ± 46.3^a^ | 111.4 ± 21.8^b^ | 251.3 ± 111.0^a,b,c^ |
| LDL-C, mg/dl | 108.2 ± 24.9 | 107.8 ± 22.3 | 179.1 ± 32.2^a,b^ | 166.6 ± 31.9^a,b^ |
| sdLDL-C, mg/dl | 27.8 ± 13.4 | 38.1 ± 16.8 | 45.4 ± 18.2^a^ | 71.7 ± 27.3^a,b,c^ |
| lbLDL-C, mg/dl | 80.4 ± 21.8 | 70.0 ± 26.0 | 133.7 ± 32.5^a,b^ | 95.0 ± 32.0^a,b,c^ |
| Non-HDL-C, mg/dl | 129.1 ± 26.4 | 141.4 ± 23.0 | 200.6 ± 33.5^a,b^ | 214.4 ± 36.3^a,b^ |
| sdLDL-C/LDL-C, % | 25.6 ± 10.5 | 36.2 ± 15.7^a^ | 25.5 ± 10.5^b^ | 43.6 ± 16.2^a,c^ |
| ApoB, mg/dl | 86.8 ± 17.7 | 96.1 ± 14.9 | 133.2 ± 23.0^a,b^ | 143.0 ± 24.8^a,b^ |
| LDL-C/apoB | 1.24 ± 0.10 | 1.12 ± 0.12^a^ | 1.35 ± 0.07^a,b^ | 1.17 ± 0.14^a,c^ |
| LDL size, nm | 259.3 ± 4.4 | 257.3 ± 4.6^a^ | 260.7 ± 4.8 | 254.9 ± 5.2^a,c^ |
| Pattern B, n (%) | 22 (19.5) | 6 (28.6) | 5 (20) | 26 (61.9) |
| sdLDL-C Q1, Q2, Q3, Q4, n | 19, 23, 33, 38 | 1, 1, 7, 12 | 0, 1, 6, 18 | 1, 0, 3, 38 |
| sdLDL-C Q1, Q2, Q3, Q4, % | 16.8, 20.4, 29.2, 33.6 | 4.8, 4.8, 33.3, 57.1 | 0, 4., 24.0, 72.0 | 2.4, 0, 7.1, 90.5 |

Data are expressed as mean ± standard deviation

Significance at p < 0.0001–0.05 by ANOVA

^a^, vs. normal; ^b^, hyper-TG; ^c^, vs. hyper-nonHDL

HDL-C: high-density lipoprotein-cholesterol, TG: triglycerides, LDL-C: low-density lipoprotein-cholesterol, sdLDL-C: small dense LDL-C, lbLDL-C: large buoyant LDL-C, Non-HDL-C: non high-density lipoprotein-cholesterol, Apo: apolipoprotein, Pattern B: LDL phenotype with an average diameter of <25.5 nm

**Table S3. Alternative LDL window in 528 patients with CAD including diabetes**

|  | “Alternative LDL window” | | | |
| --- | --- | --- | --- | --- |
| Groups | Normal | Hyper-TG | Hyper-nonHDL | Hyper-TG/-non-HDL |
| Number (M/F)  %N/total N | 317 (250/67) | 80 (67/13) | 58 (40/18) | 73 (57/16) |
|  | 60.0 | 15.2 | 11.0 | 13.8 |
| Total cholesterol, mg/dl | 180.5 ± 26.6 | 189.7 ± 20.0^a^ | 239.1 ± 25.2^a,b^ | 244.3 ± 26.0^a,b^ |
| HDL-C, mg/dl | 49.7 ± 14.5 | 43.0 ± 12.6^a^ | 46.4 ± 10.7 | 41.4 ± 8.4^a^ |
| TG, mg/dl | 92.0 ± 28.3 | 215.6 ± 78.0^a^ | 106.2 ± 31.6^b^ | 230.8 ± 78.4^a,c^ |
| LDL-C, mg/dl | 110.6 ± 24.4 | 111.8 ± 21.9 | 168.6 ± 25.0^a,b^ | 159.8 ± 30.1^a,b^ |
| sdLDL-C, mg/dl | 26.7 ± 13.8 | 40.6 ± 16.6^a^ | 45.3 ± 25.3^a^ | 58.9 ± 20.3^a,b,c^ |
| lbLDL-C, mg/dl | 80.1 ± 23.2 | 65.7 ± 21.1^a^ | 117.9 ± 32.7^a,b^ | 96.6 ± 31.0^a,b,c^ |
| Non-HDL-C, mg/dl | 130.7 ± 21.5 | 146.7 ± 17.9^a^ | 192.8 ± 21.3^a,b^ | 202.8 ± 23.3^a,b,c^ |
| sdLDL-C/LDL-C, % | 25.5 ± 13.3 | 38.5 ± 14.9^a^ | 28.0 ± 15.3^b^ | 38.9 ± 13.9^a,c^ |
| ApoB, mg/dl | 85.9 ± 15.3 | 97.1 ± 13.9^a^ | 123.5 ± 15.4^a,b^ | 131.5 ± 18.5^a,b,c^ |
| LDL-C/apoB | 1.24 ± 0.17 | 1.10 ± 0.13^a^ | 1.33 ± 0.13^a,b^ | 1.18 ± 0.15^a,b,c^ |
| LDL size, nm | 257.5 ± 3.6 | 252.9 ± 4.2^a^ | 256.6 ± 3.3^b^ | 253.9 ± 4.1^a,c^ |
| Pattern B, n (%) | 111 (35.0) | 65 (81.3) | 25 (43.1) | 56 (76.7) |
| sdLDL-C Q1, Q2, Q3, Q4, n | 63, 75, 8, 98 | 3, 4, 23, 50 | 1, 5, 13, 39 | 0, 1, 3, 69 |
| sdLDL-C Q1, Q2, Q3, Q4, % | 19.9, 23.7, 25.6, 30.9 | 3.8, 5.0, 28.8, 62.5 | 1.7, 8.6, 22.4, 67.2 | 0, 1.4, 4.1, 94.5 |

Data are expressed as mean ± standard deviation

Significance at p < 0.0001–0.05 by ANOVA

^a^, vs. normal; ^b^, hyper-TG; ^c^, vs. hyper-nonHDL

HDL-C: high-density lipoprotein-cholesterol, TG: triglycerides, LDL-C: low-density lipoprotein-cholesterol, sdLDL-C: small dense LDL-C, lbLDL-C: large buoyant LDL-C, Non-HDL-C: non high-density lipoprotein-cholesterol, Apo: apolipoprotein, Pattern B: LDL phenotype with an average diameter of <25.5 nm
